# A BEST example of channel structure annotation by molecular simulation

**DOI:** 10.1080/19336950.2017.1306163

**Published:** 2017-03-20

**Authors:** Shanlin Rao, Gianni Klesse, Phillip J. Stansfeld, Stephen J. Tucker, Mark S. P. Sansom

**Affiliations:** a Department of Biochemistry, University of Oxford, Oxford, UK; b Clarendon Laboratory, Department of Physics, University of Oxford, Oxford, UK

**Keywords:** free energy, hydrophobic gating, molecular dynamics, water

## Abstract

An increasing number of ion channel structures are being determined. This generates a need for computational tools to enable functional annotation of channel structures. However, several studies of ion channel and model pores have indicated that the physical dimensions of a pore are not always a reliable indicator of its conductive status. This is due to the unusual behavior of water within nano-confined spaces, resulting in a phenomenon referred to as “hydrophobic gating”. We have recently demonstrated how simulating the behavior of water within an ion channel pore can be used to predict its conductive status. In this addendum to our study, we apply this method to compare the recently solved structure of a mutant of the bestrophin chloride channel BEST1 with that of the wild-type channel. Our results support the hypothesis of a hydrophobic gate within the narrow neck of BEST1. This provides further validation that this simulation approach provides the basis for an accurate and computationally efficient tool for the functional annotation of ion channel structures.

## Introduction

Ongoing advances in the determination of ion channel structures now clearly necessitate the development of accurate computational tools to aid the functional annotation of both new and known structures.^1^ One critical aspect of such annotation concerns the likely ion conductive state represented by these different structures and conformations, i.e. we require methods for identifying whether a given structure represents an open state of the channel, which supports rapid ion permeation, or a closed non-conductive state (which in this context also includes desensitized and/or inactivated states).

To date one of the most widely used methods for predicting whether a channel structure is conductive or not has been to evaluate the physical dimensions of the pore, often via the use of HOLE^2^ to generate an image of the pore-lining surface and/or calculate a pore radius profile. The radius profile is then compared with the known radius of a hydrated ion to determine whether the channel is likely to be closed or open. However, this approach does not consider the nature of the pore lining, and specifically whether the lining of a narrow pore is sufficiently hydrophobic to form a functionally closed pore. This is important in the context of the model of *hydrophobic gating*.^3-6^ Thus, even when large enough to accommodate ions, hydrophobic regions with radii below ∼5 Å may present a significant energetic barrier to the flow of water and ions through a pore. Such cases of hydrophobic gating are associated with the phenomenon of de-wetting, whereby the presence of liquid-phase water is unfavorable within a narrow hydrophobic pore, leading to formation of a ‘vapor lock’ (i.e., a region of the pore not occupied by water molecules). It should be noted that at the nanoscale (e.g. radius less than 5 Å) an ‘empty’ region of the pore corresponds to a vapor phase, as the number of water molecules in the vapor in such a volume would be << 1. This model of gating was originally proposed based on simplified models of nanopores,^3,7^ and nanotubes.^8^ The mechanism has since received computational and experimental validation for several ion channel proteins.^6,9^ Thus a functionally open or closed state cannot simply be assigned to a channel structure solely by assessing the physical dimensions of the pore, but rather the relative hydrophobicity of these pores and the behavior of water within them should also be considered.

In a recent paper^1^ we presented a molecular dynamics simulation-based approach for the improved functional annotation of ion channels. Three alternative levels of simulation were compared, at increasing levels of computational complexity: (i) simulations of the equilibrium distribution of water within the channel pore; (ii) potential of mean force (PMF) calculations that yield free energy profiles for single ions along the pore axis; and (iii) computational electrophysiology,^10^ whereby a membrane potential is imposed on an ion channel in a bilayer and ion flux is simulated directly. Of these the first is the simplest and the least computationally demanding, and therefore is amenable to automated annotation of novel channel structures. Thus, the equilibrium distribution of water molecules within a pore is taken as a “proxy” for whether or not a pore can conduct ions.

In the previous paper^1^ the method was evaluated via its application to members of the pentameric ligand-gated ion channel (pLGIC) family, as there have been several previous studies suggesting that hydrophobic gating is found in pLGICs.^11,12^ In particular, a recent structure of the serotonin receptor (5HT3R) and different conformations of the glycine receptor (GlyR) were examined. We therefore wished to apply the same approach to an unrelated family of ion channels which may also contain a hydrophobic gate.

Eukaryotic bestrophins are calcium-activated chloride channels with a broad tissue distribution.^13^ Bestrophin-1 (BEST1) is highly expressed in the retinal pigment epithelium, and dysfunction of the human channel has been associated with macular degenerative disorders.^14^ Crystal structures of chicken BEST1^15^ and of its bacterial (*Klebsiella pneumoniae*) homolog KpBest^16^ have been determined and thus also provide an opportunity to further assess the applicability of this new approach.

**Figure 1.**
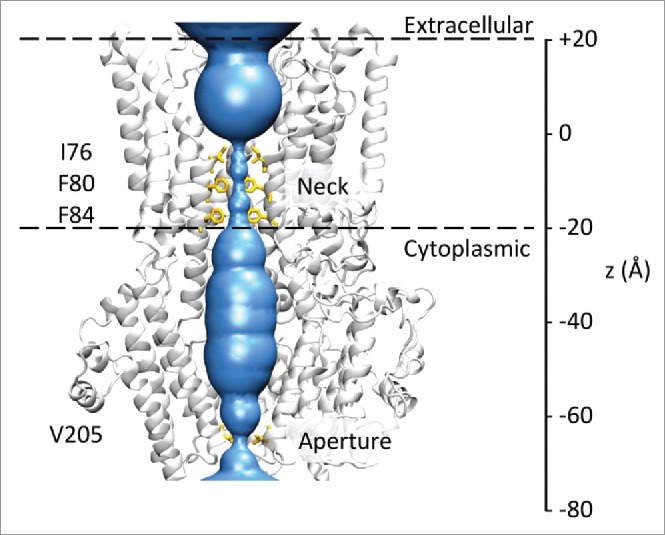
Structure of the BEST1 channel. The protein fold of 3 of the 5 subunits of the channel is shown in gray, with the pore lining surface (calculated using HOLE) in blue. Hydrophobic side-chains lining the neck and aperture regions of the pore are shown as sticks in yellow. The 2 broken horizontal lines indicate the approximate location of the membrane. The bilayer normal and pore axis correspond to the z-axis shown on the right.

The BEST1 channel is a symmetric assembly of 5 subunits surrounding a central pore (Fig. 1). There is a wide extracellular mouth and vestibule leading into a narrow ‘neck’ region lined by 3 rings of conserved hydrophobic amino acids: Ile76, Phe80, and Phe84. The pore then widens as it passes through the cytoplasmic domain before reaching an additional constriction (the “aperture”) formed by a ring of valine residues (Val205).^13,15^

Interestingly, the narrow neck region has been implicated in the gating mechanism of BEST1. A structure has been determined for a “triple A” mutant, in which the 3 bulky hydrophobic residues lining the neck were replaced by alanine.^13^ Ionic current measurements and chloride flux assays on liposome-reconstituted channels demonstrated that widening of the neck by this triple A mutation increased chloride transport rates. Thus, as the wild-type (BEST1_WT_) and triple alanine mutant (BEST1_AAA_) structures differ in the radius of the proposed gate in the hydrophobic neck region, this channel structure provides an excellent test for further examination of this method for the functional annotation of ion channel structures.

## Results and discussion

### De-wetting of BEST1 channel pore at the hydrophobic gate in the neck region

**Figure 2.**
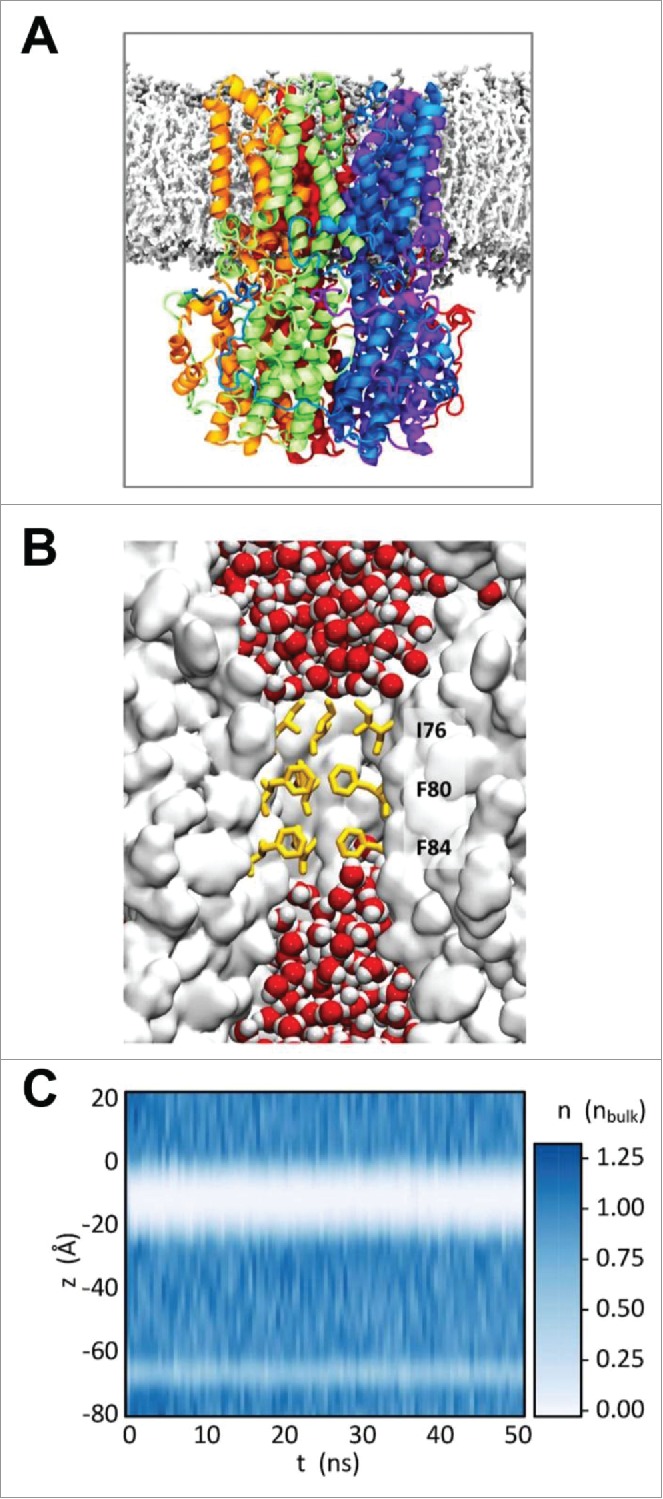
De-wetting of the hydrophobic neck region within the pore of BEST1. (A) Simulation system of BEST1_WT_ (PDB: 4RDQ) embedded in a POPC lipid bilayer. A ribbon representation of the pentameric protein is colored by subunit. Phospholipid headgroups are colored gray and the glycerol backbone and acyl chains in white. Surrounding water molecules and ions are omitted for clarity. (B) Zoomed-in view of the neck region of the central pore, showing water molecules (red/white) at each mouth of the neck. Sidechains (I76, F80, and F84) forming the hydrophobic gate in the neck are shown in yellow. (C) Water density within the channel along the pore axis as a function of simulation time, normalized to the average density of bulk water.

The BEST1 structure was embedded *in silico* within a phospholipid (POPC, i.e., 1-palmitoyl-2-oleoyl-*sn*-glycero-3-phosphocholine) bilayer (Fig. 2A), with water molecules as well as Cl^−^ and Na^+^ ions (at a concentration of 0.15 M) on either side. To retain the exact conformation of the experimentally determined structure, the positions of the protein atoms were restrained. Upon simulation at 37°C, water molecules were observed within the pore (Fig. 2B) and their time-dependent density as a function of position along the pore (z) axis (Fig. 2C) was then analyzed.

**Figure 3.**
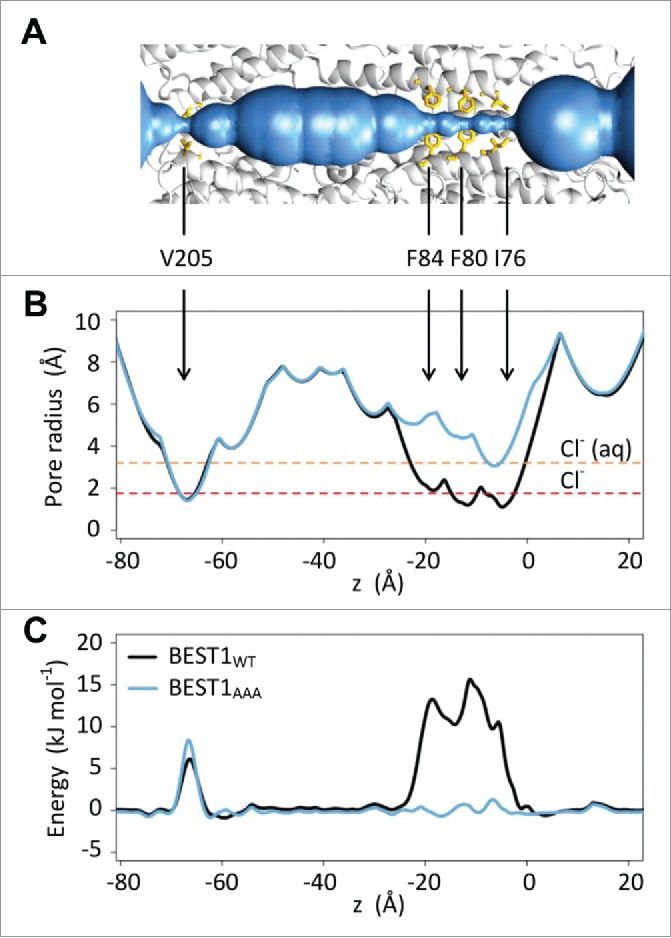
Pore radius and water permeation free energy profiles (A) HOLE profile of the central pore of the BEST1_WT_ channel structure, alongside a ribbon representation (gray) of 3 adjacent subunits of the protein. Hydrophobic neck (I76, F80, and F84) and aperture (V205) residues are shown in yellow. (B) Pore radius profiles for BEST1_WT_ (black line) BEST1_AAA_ (blue line) channel structures. Red and orange dashed lines indicate the radii of a dehydrated and hydrated chloride ion respectively. (C) Free energy profile of a water molecule along the z-axis through the pores of BEST1_WT_ (black) and BEST1_AAA_ (blue). Positional restraints were applied to all protein atoms throughout these simulations.

The approximately 1.5–2 Å radius at the neck region of the BEST1_WT_ structure is comparable to that of a dehydrated chloride ion (Fig. 3A and B). The neck had previously been suggested to be able to accommodate passage of single ions,^15^ although more recent studies suggest it plays a role in gating and may instead correspond to a closed or inactivated state of the channel.^13^ In the simulations, 5 or 6 water molecules that were initially present in the neck are rapidly expelled on a sub-nanosecond time scale. The hydrophobic neck (approximately 16 Å in length) then remains predominantly de-wetted over the course of a 50 ns simulation. This low density of water within the neck (Fig. 2C) contrasts with the water distribution in other regions of the central pore where water molecules remain throughout the duration of our simulations.

The water density may be used to estimate the free energy profile for water along the axis of the pore (Fig. 3C; see Methods below for details). This reveals that there is an energy barrier in the neck region of 10–15 kJ mol^−1^ (this should be compared with a mean thermal energy of ∼2.6 kJ mol^−1^ at physiologic temperature). This structure of BEST1 can therefore be clearly assigned to a closed, non-conductive conformation. There is also a smaller energy barrier in the vicinity of the V205 aperture.

### Wild-type and mutant structures reveal different functional states

The triple alanine mutant (BEST1_AAA_) structure of the channel appears to correspond to a constitutively open state^13^ and comparison of the pore radius profiles appears to support this suggestion (Fig. 3B); the pore radius is increased from 1.5–2 Å in the wild-type channel to > 3 Å in the mutant channel. Furthermore, our observation that the large energetic barrier to water permeation in the neck is removed in the mutant structure, whereas the smaller barrier in the aperture region remains unchanged supports the suggestion that the neck forms a hydrophobic gate in BEST1. Thus, by using water permeation free energy as a proxy for channel conductance, the BEST1_AAA_ structure is predicted to have a functionally open gate. Interestingly, the results provide a more complex example of a hydrophobic gating motif than previously observed in e.g., the L9' sidechain ring of pLGICs and further demonstrate the capability of our simulation method to allow annotation of conductive state of new ion channel structures.

### Robustness of the annotation method

**Figure 4.**
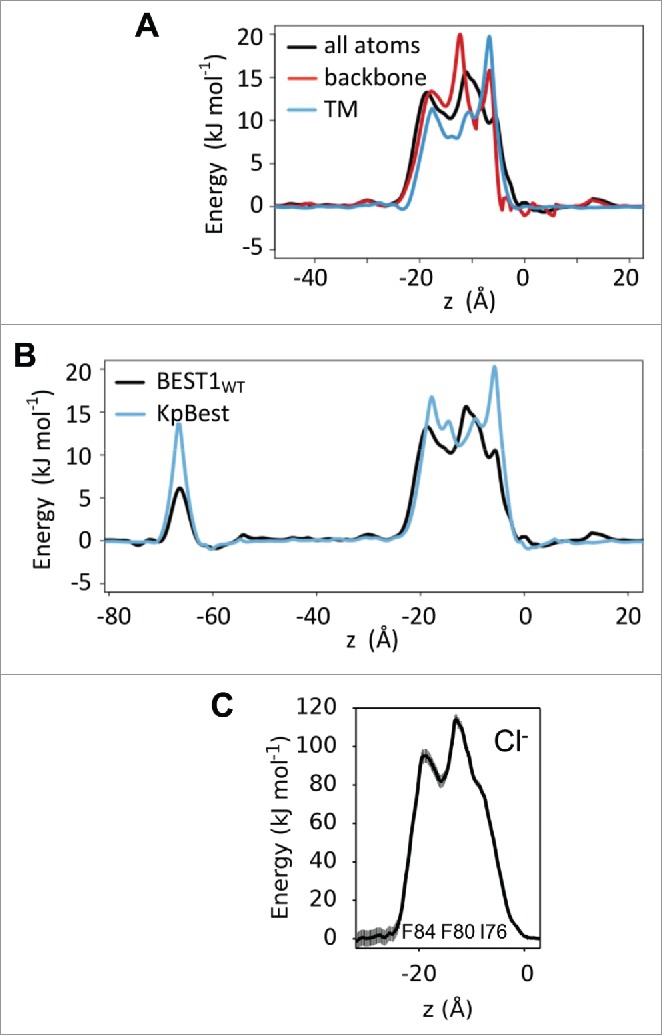
Robustness of the approach. (A) Comparison of the water permeation free energy profiles for the neck region of BEST1_WT_ channel for simulations in which all atoms were restrained (black), only backbone atoms were restraints (red), and when only the transmembrane (TM) helices were simulated (blue). (B) Comparison of water free energy profiles for the chicken BEST1_WT_ channel and its bacterial homolog KpBest (PDB: 4WD8). In both cases, all protein atoms were restrained during these simulations. (C) Potential of mean force (PMF) calculations for a Cl^−^ ion as a function of position along the axis of pore in the neck region of the BEST1_WT_ channel (derived from simulations where only the TM helices were present).

In our simulations the channel conformation was preserved by imposing restraints on the structure. However, at room temperature thermal fluctuations might be expected to permit dynamic changes in the conformation of side-chains lining the channel. Permeation of water and ions through a channel is influenced by the local flexibility of the pore lining, as demonstrated in earlier studies of hydrophobic gating.^5^ To address this issue, the BEST1_WT_ structure was also simulated with restraints applied only to the protein backbone, thereby allowing a degree of (local) side-chain flexibility. However, despite the increased degree of conformational mobility and resulting shifts in pore radii (changes of < 1 Å), the large energetic barrier to water permeation within the neck remained (Fig. 4A). Furthermore, we examined whether simulating just the transmembrane (TM) domain of BEST1_WT_ embedded in a POPC bilayer yielded free energy profiles comparable to those obtained from simulations of the full-length channel. Fig. 4A shows similar results were obtained for this reduced system suggesting that the functionally closed state is an intrinsic property of the TM domain (and of the neck) rather than a property of the pore as a whole. As a further test of the robustness of the simulation results to variation in the input structure, the homologous KpBest channel structure (which has the same overall fold and conformation as the eukaryotic BEST1 structure^16^) was simulated and found to generate a very similar energetic profile for water as the BEST1_WT_ (Fig. 4B).

We have also tested the robustness of the assumption underlying the use of simulations of water within the pore of an ion channel as a proxy for the energetics of ion permeation. We used umbrella sampling simulations^17^ to estimate the PMF (i.e., free energy profile) for a chloride ion as a function of its position along the pore axis of the BEST1 TM domain within a lipid bilayer. This revealed that the neck region of BEST1_WT_ presents a considerable energetic barrier (∼100 kJ mol^−1^) to chloride ion permeation (Fig. 4C). Furthermore the profile of this energetic barrier corresponds closely to that observed for water, with 3 peaks corresponding to the 3 rings of hydrophobic side-chains which line the neck region of the pore.

### Concluding remarks

We have demonstrated the utility of a simulation-based annotation approach to determine the likely conductive state of a given ion channel structure as applied to BEST1. We have also examined the robustness of the method with respect to minor changes in the simulation protocol, such as the use of different conformational restraints and examination of the TM-only domains. Thus, combined with the results of our previous study,^1^ this now demonstrates the overall utility of the approach and provides the basis for future refinement and automation of this simulation-based tool for the functional annotation of ion channel structures. Furthermore, the results extend the range of hydrophobic gating motifs beyond that of e.g., the L9' side-chain ring of pLGICs.

## Methods

### Molecular dynamics simulations

Starting from the experimentally determined protein structure (PDB ID 4RDQ for the wild-type; 5T5N for the triple alanine mutant), the simulation system was prepared using a sequential multi-scale procedure as described previously.^18^ Briefly, the protein structure was first coarse-grained and then simulated for 50 ns together with coarse-grained POPC lipids, water particles, and sodium and chloride ions using version 2.2 of the Martini force field,^19,20^ and the Gromacs^21^ molecular dynamics package v5.1. During the simulation, lipid molecules self-assembled into a bilayer around the protein. It was checked that none blocked the permeation pathway. The resulting protein-bilayer system was subsequently converted back to its atomistic representation and simulated for a further 100 ns using the TIP4P water model^22^ and OPLS united atom force field.^23^ Long range electrostatics were treated using the particle-mesh Ewald (PME) algorithm,^24^ using a real-space cutoff of 1 nm and a Fourier spacing of 0.12 nm. The system was maintained in the isothermal-isobaric (NPT) ensemble at a temperature of 310 K and a pressure of 100 kPa using a v-rescale thermostat^25^ with a coupling constant of 0.1 ps and a semi-isotropic Parinello-Rahman barostat^26^ with a coupling constant of 1 ps. Time integration was performed using the canonical leapfrog method with a time step of 2 fs and the LINCS algorithm^27^ was used to constrain bonds. Additionally, positional restraints (force constant 1000 kJ nm^−2^ mol^−1^) were applied to either to all protein atoms or to the backbone atoms. Post-processing of the simulation trajectories was performed using Gromacs. Molecular graphics were created with VMD.^28^

### Boltzmann inversion and free energy profiles

Since the atomistic simulations reflect equilibrium conditions, the number density of water molecules at a given position along the main axis of the BEST1 pore, *n(z)*, is related to the free energy at this position, *E(z)*, through

$$n\left(z\right)=Cexp\left(\frac{-E\left(z\right)}{kT}\right)$$

where *z* is the Cartesian coordinate normal to the plane of the membrane, *k* is Boltzmann's constant, *T* is absolute temperature, and *C* is a normalization constant related to the system's partition function. Inverting the above relation yields

E (z)=−kTln[n(z)]−kTln[C]

and it becomes apparent that C is effectively an additive constant independent of *z*, which we determine by setting the free energy in the bulk outside the pore to zero. The number density of water particles can be determined by dividing the water count at a given position by the local cross-sectional area of the pore (approximated as circular) and calculated using HOLE.^2^

### Umbrella sampling and PMF calculation

Umbrella sampling simulations were initiated using an equilibrated atomistic system containing the BEST1 TM domain embedded in a lipid bilayer and surrounded by water and ions, as described above. In 36 consecutive sampling windows, a single chloride ion was placed at successive z positions along the central pore axis, spaced at 1 Å intervals. The sampling windows covered the neck region and extended approximately 10 Å either side. A harmonic biasing potential was applied to the *z* coordinate of the chloride ion with a force constant of 1000 kJ mol^−1^ nm^−2^. Each window was simulated for 5 ns, during which positional restraints (force constant 1000 kJ mol^−1^ nm^−2^) were applied to backbone atoms of the protein. PMFs were computed using the weighted histogram analysis method (WHAM), and errors on each free energy profile were estimated using the Bayesian bootstrapping method.^29^ Convergence was assessed by comparing free energy profiles computed from consecutive fractions of simulation time.
